# Barriers experienced by people with disabilities participating in income-generating activities. A case of a sheltered workshop in Bloemfontein, South Africa

**DOI:** 10.4102/ajod.v9i0.662

**Published:** 2020-08-31

**Authors:** Nokuthula Tinta, Hester Steyn, Jana Vermaas

**Affiliations:** 1 Department of Consumer Science, Faculty of Natural and Agricultural Sciences, University of the Free State, Bloemfontein, South Africa

**Keywords:** people with disabilities, barriers, income generating activities, sheltered workshop, participation

## Abstract

**Background:**

People with disabilities often participate in income-generating activities (IGAs) in sheltered workshop in South Africa. However, they face many barriers that limit their ability to participate effectively in economic activities hosted by the workshops.

**Objectives:**

To illustrate the barriers that limit the participation of people with disabilities in IGAs in a sheltered workshop.

**Method:**

A qualitative exploratory single case study was conducted in a sheltered workshop. Eighteen participants, age 22 to 52 years with various disabilities were purposively sampled. Observations and semi-structured interview guides were used to generate data. Verbatim transcription was used after which content analysis was applied to identify ideas and concepts relating to barriers experienced by people with disabilities participating in IGAs.

**Results:**

Some of the barriers participants experienced included institutional barriers (ability to use working tools, inability to concentrate for long periods, lack of funds, language barriers, lack of motivation, activities that are not stimulating and lack of artistry skills) and attitudinal barriers (exclusion from decision making) These barriers had an adverse influence on their performance in IGAs.

**Conclusion:**

The study found eight different barriers that existed in a sheltered workshop which limited the participation of the people with disabilities that attended the workshop. This information can be used to develop strategies to address each barrier and promote increased participation of the individual thereby improving their quality of life.

## Introduction

Over 15% of the world’s population is made up of people with disabilities (World Health Organization & World Bank [WHO & WB] 2011), and these people are often unemployed, live in poverty, and some may have no qualifications. South Africa has a population of 57.73 million of which 2.9 million (7.5%) are recorded as living with a disability and experience difficulties in functioning and participating in economic activities (Stats SA 2014). Consequently, this has a negative impact on their lives. The South African government developed various policy frameworks and guidelines since 1994. These include the Skills Development Act No. 97 of 1998 (Republic of South Africa 1998b), National Skills Development Strategies (NSDS, Republic of South Africa 2001, 2005, 2011), White Paper on an Integrated National Disability Strategy (INDS, Republic of South Africa 1997b), Code of Good Practice on the employment of people with disabilities (Republic of South Africa 2002), Promotion of Equality and Prevention of Unfair Discrimination (PEPUDA, Republic of South Africa 2000), and the Employment Equity Act 55 (EEA, Republic of South Africa 1998a). These were developed to enable the successful inclusion of people with disabilities into mainstream society. These policies and frameworks aim to increase access and opportunities to ensure that all people can develop livelihoods that are sustainable.

Additionally, the policies deal with the challenges that limit the ability of people with disabilities to participate in society. They are also intended to address the needs of those people who were previously disadvantaged as well as marginalised groups like people with disabilities. Despite these policies, guidelines and frameworks, it is argued that people with disabilities in a sheltered workshop (a setting in which people with disabilities receive services and training to develop work-related skills and behaviours, Armstrong 2011) are faced with attitudinal, environmental and institutional barriers, which limit their ability to participate in economic activities effectively. Research shows that people with disabilities in South Africa face interacting barriers that limit life chances and their ability to participate in economic activities. These barriers include lack of accessible transport to reach the workplace, lack of skills training, lack of application and monitoring of the Employment Equity Act and lack of funds (Berthoud 2008; Department of Labour 2002; Economic Policy Research Institute [EPRI] 2001, 2004; Moodley 1997; Seirlis & Swartz 2006). A study by Momin (2004) reveals that people with disabilities suffer from various forms of barriers such as appropriate skills, lack of educational qualifications, discriminating attitude and inappropriate wages. Literature also shows that different impairment characteristics, as well as the type of disability, also influence participation (Berthoud 2008; Jones & Jones 2008; Meager & Higgins 2011). Such barriers are over and above those faced by people without disabilities, and as a result, people with disabilities experience difficulty in accessing education or have lower educational achievement and are economically inactive (Hoogeveen 2005; Rischewski et al. 2008; Mont & Viet Cuong 2011). This exclusion results in people with disabilities working in sheltered employment which, in South Africa, is offered by the state or by private welfare organisations and self-help programmes, which are not sustainable in themselves. Sheltered employment across Europe is one of the most broadly used employment measures for people with disabilities (Mallender et al. 2015). Several studies have reported growth in sheltered workshop placement in Germany and Spain (Flores et al. 2011; Shima, Zólyomi & Zaidi 2008).

However, little is known in South Africa about the challenges people with disabilities who participate in income-generating activities (IGAs) experience. There is also a dearth of information on whether people with disabilities are being prepared for the open labour market. The objective of this paper was to illustrate the barriers that limit the participation of people with disabilities in IGAs in a sheltered workshop.

## Literature review

Disability is the broad term that covers impairments such as movement impediment and limitation to participate. These impairments restrict the body to function appropriately or affect the structure of the body.

Disability, therefore, limits individuals from performing activities or participating in day-to-day life situations (WHO & WB 2011). In South Africa, people with disabilities are classified into six categories based on the degree of difficulty they experience. The categories used for the 2011 Census are seeing, hearing, communication, physical, mental and difficulty in self-care (Stats SA 2014). Based on the disability types, one can contend that there is a range of particular needs that should be met to ensure that the productivity of individuals is maximised for each disability type. The social model of disability of Gottlieb, Myhill and Blanck (2010), which is increasingly recognised by various researchers (Opoku et al. 2017; Thibedeau Boyd & Davis 2016; Walsh, Lydon & Healy 2014), acknowledges that disability is socially constructed. This means that the cause of the ‘problem’ is placed on society rather than on the individual with impairment. Conversely, the medical model sees the physical limitation as the primary cause of disadvantage for a disabled person. The social model does not deny that a person with a disability might have functional limitations caused by the impairment. Rather the model evaluates the socio-economic environment as well as the impact of the barriers on the full participation and inclusion of people with disabilities as part of mainstream society. The social model recognises people with disabilities as equal citizens with full political, social, economic and human rights.

This model also emphasises the need for attitude changes in society with the provision of accessible services and activities, and the mainstreaming of disability to ensure inclusivity and equality (Republic of South Africa 2017). The model further gives prominence to people with disabilities to participate actively in transformation processes to improve the quality of their lives. Unlike the medical model, which disseminates sheltered employment opportunities that are not part of the open labour market, the social model has had positive outcomes for employment in countries like the United States, Canada and Australia (Gottlieb et al. 2010). In the view of Shapiro (1994), this has helped change employers’ perceptions of people with disabilities. People with disabilities may face environmental barriers whereby physical environment, transportation, technology, informational sources and buildings marginalise them. For people with disabilities to participate fully in community life, access to these environmental surroundings and facilities is essential (Braithwaite & Mont 2008). Metu (2011) studied factors influencing the performance of IGAs among persons with physical disabilities. The author found that the majority had high levels of physical and environmental barriers affecting their mobility and subsequently, their choice of business and ability to run it.

Yeo and Moore (2003) further point out that the scarcity of information transcribed into Braille as well as the lack of sign language interpretation for those with sensory impairments is among the barriers that keep people with disabilities from participating fully. Barriers may occur in institutions. Institutional barriers for people with disabilities exist when they are discriminated against in various ways or fail to take full account of their needs (Barnes 1991). Research shows that there is insufficient learning material to teach children with disabilities in South Africa (Fish-Hodgson & Khumalo 2015). Barriers may also be attitudinal.

Misconceptions about the ability of people with disabilities to perform jobs are one of the main reasons for their exclusion from opportunities (Shier, Graham & Jones 2009). This may come from the belief that people with disabilities are less productive than their non-disabled equivalents. People with disabilities are also regarded as not being able to contribute anything, are waiting to be helped and are dependent on others (Uromi & Mazangwa 2015). Discrimination against people with disabilities often stems from a lack of knowledge and negative attitude. Naami (2014) states that a person with disabilities experiences discrimination daily in all spheres of life, because of ignorance and misconceptions about their capabilities. Marumoagae (2012) points out that discrimination against people with disabilities is one of the worst social injustices that society has not been able to overcome. Some disabilities can cause more severe discrimination than others. Baldwin and Johnson (1994), as well as Baldwin and Marcus (2006), point out that different impairments can cause different degrees of prejudgement, with the most substantial prejudice exhibited towards people with mental health conditions. The barriers discussed also impose severe limits on life chances for specific groups of people with disabilities. The EEA (Republic of South Africa 1998a) states that it is the responsibility of employers to accommodate people with disabilities. However, the EEA (Republic of South Africa 1998a) does not provide a clear definition of what constitutes a reasonable accommodation. This is most likely because the type of accommodation and related processes depend on the nature of the disability. The Code of Good Practice provides certain instances of what can be considered reasonable accommodation (Republic of South Africa 2002). These include adapting facilities, adapting equipment or acquiring new equipment, re-organising work changing training and assessment materials and systems, restructuring jobs, providing readers with sign language interpreters, and providing specialised supervision, training and support. Several studies (Bruyere, Erickson & VanLooy 2000; Cleveland, Barnes-Farrell & Rats 1997; Lee 1996; Mitchell, Alliger & Morfopoulos 1997) investigated the types of reasonable accommodation implemented by employers. Smith, Poval and Floyd (1991) suggested guidelines to increase an understanding of disability in the workplace. These scholars recommend that special disability training be provided to a variety of staff within an organisation. These include staff who are involved in training, staff formally responsible for disability issues, staff involved in selection and recruitment, managers with newly appointed disabled employees, and staff with disabilities.

## Research method and design

### Study design

A qualitative research design was chosen for this exploratory, single case study, which involves the collection, organisation and interpretation of data gained through interviews and observations (Malterud 2001). An exclusive qualitative method is effective when little is known about a topic and is a ‘strong initial research option’ (Hartley & Muhit 2003), as is the case of this study where the challenges people with disabilities faced in South African IGAs were explored.

### Setting

The study was conducted in one of the sheltered workshops in Bloemfontein, Free State (South Africa). This workshop is under the management and supervision of the Association for People with Disabilities, which is a non-profit organisation. The sheltered workshop consists of one manager, one facilitator and 54 people with disabilities operating in different groups. They generate income through handmade products sold to the public and businesses.

### Study population and sampling strategy

The study population during the period of the study consisted of 54 people with disabilities. A total of 24 participants were selected for the study using purposeful sampling and included as much diversity as possible.

Men and women of working age (18–60 years), representing diverse ethnic groups and languages such as Xhosa, Zulu, Sotho, Afrikaans, and Tswana were selected. Participants with physical, mental and intellectual disabilities who had been participating in IGAs in the sheltered workshop for longer than 6 months and who are recipients of a disability grant were included. Participant detailed demographic characteristics are displayed in Table 1.

**TABLE 1:** Demographic profile of the participants.

Pseudonym	Gender	Age	Marital status	Home language	Population group	Disability type	Level of education	Length of time in the workshop
A	Female	29	Single	Sesotho	Black	Epilepsy	Grade 8	5 years
B	Male	25	Single	isiZulu	Black	Epilepsy	Grade 9	6 years
C	Male	36	Single	Setswana	Black	Dyslexia	Grade 7	8 years
D	Male	43	Single	Setswana	Black	Lame right limbs (stroke)	Grade 9	23 years
E	Female	46	Single	Afrikaans	Mixed race	Learning and lame right limbs	Grade 7	17 years, 4 months
F	Male	28	Single	isiXhosa	Black	Bone and joint deformities and sight	Grade 7	11 years
G	Female	26	Single	Afrikaans	Mixed race	Spinal Bifida	Grade 9	4 years
H	Male	24	Single	Sesotho	Black	Cerebral palsy	Grade 7	3 years
I	Female	44	Single	isiXhosa	Black	Involuntary muscle movement (both legs)	No schooling	9 years
J	Female	36	Single	isiXhosa	Black	Epilepsy	Grade 7	20 years
K	Female	24	Single	isiXhosa	Black	Right limbs lame	Grade 7	Cannot remember
L	Female	22	Single	Afrikaans	Mixed race	Physical (unknown)	Grade 9	1 year, 4 months
M	Male	27	Single	Sesotho	Black	Left limbs lame and learning	Grade 11	3 years
N	Male	22	Single	Sesotho	Black	Lame right limbs (stroke)	Grade 9	3 years
O	Female	52	Single	isiXhosa	Black	Epilepsy and sight	Grade 2	Cannot remember
P	Female	38	Single	Sesotho	Black	Down syndrome	Grade 7	20 years
Q	Male	46	Widower	Sesotho	Black	Lame left hand (stroke)	Grade 7	10 years
R	Female	43	Single	Sesotho	Black	Learning and epilepsy	No schooling	9 years

### Data collection

Observations and semi-structured interviews were used to collect data between August and September 2016.

The researcher obtained permission to conduct the study from the manager of the workshop before the study commenced. The researcher established rapport with the participants as one of the strategies to obtain approval and assured them confidentiality (eds. Ritchie et al. 2013). The researcher visited the workshop and took notes. Observations were conducted four times a week, for an hour and a half per day for 2 months. According to Creswell (2007, 2013), prolonged engagement with participants ensures that false conclusions were not reached. The observational protocol captured information about the challenges experienced by participants while making handmade products. Field notes were used to report and reflect on everything that was observed in the workshop.

After observations, individual interviews were arranged with participants. The interviews comprised short questions which evoked responses that reflected participants’ experiences with IGAs.

Interview sessions, which lasted an hour each, were digitally recorded. The participants agreed that all the interview sessions could take place at the sheltered workshop as it was convenient for them. The interview schedule was constructed in English, translated into Sesotho and isiXhosa and administered in the same languages. The reason for translating this interview schedule in these languages was that the majority of the participants in the sheltered workshop spoke Sesotho and isiXhosa. The researcher is fluent in isiXhosa, isiZulu and English, but had difficulties in comprehending Sesotho. Thus an assistant was enlisted who was fluent in Sesotho and English. The researcher trained the assistant on how to conduct interviews and feedback meetings were held to clarify issues and ensure that there was a shared understanding of the data gathered.

Data collection ceased after data saturation was reached. The researcher, therefore, conducted interviews with 18 of the 24 participants selected for the study. In qualitative studies, 12–15 participants are generally considered sufficient for a study (Green & Thorogood 2004). The researcher returned to the participants with the transcribed interviews. They were satisfied with the content and indicated that they had nothing to add.

### Data analysis

Recorded data was transcribed in the original languages. The transcribed data was then translated into English and then transcribed verbatim before analysis was done. Two bilingual individuals checked the quality of the translations. Inductive content analysis was applied (eds. Ritchie et al. 2013). Content analysis was selected because it is a widely used qualitative research technique and researchers regard it as a flexible method for analysing text data (Cavanagh 1997). To gain familiarity with the text, the researcher read and noted down initial ideas. Ideas and concepts were identified, and relevant units were coded, which were then clustered into subcategories. Paradoxes and conflicting ideas were explored, and similar opinions were merged (eds. Onwuegbuzie et al. 2009; Ritchie et al. 2013). The researcher finally interpreted the findings. The number of participants was small; thus data was analysed manually (Gobo 2008). Verbatim quotations from interview transcripts were extracted to illustrate relevant themes where appropriate.

### Ethical consideration

Trustworthiness was established according to the recommended strategies from published methodological sources (Lincoln & Guba 1985). No incentives were offered, increasing the possibility that participants had an interest in the topic. A pilot study was done to eliminate possible errors and problems in the research instruments. Ethical clearance was obtained from the Faculty of Natural and Agriculture Science Ethics Committee, University of the Free State, reference number: UFS-HSD2016/1436. Verbal consent was obtained from all participants before and during data collection in the presence of a facilitator. Participation was voluntary, and participants could withdraw any time they wanted to. Confidentiality was maintained throughout the study by using pseudonyms on audio recordings, interview notes, and transcripts.

## Findings and discussions

The main theme identified was ‘experiences of people with disabilities in the workshop’ Four subthemes were identified in the analysis of the findings. For the purpose of this article only two subthemes will be focused on, namely, barriers experienced with IGAs and coping strategies to overcome the barriers. Other themes will be discussed at a later stage because of the amount of information and the length of the article.

### Barriers experienced with income-generating activities

Participants identified several barriers that limited their participation in economic activities. The participants reported on institutional barriers (ability to use working tools, inability to concentrate for long periods, lack of funds, language barriers, lack of motivation, activities not stimulating, lack of artistry skills) and attitudinal barriers (exclusion from decision making).

### Institutional barriers

#### Ability to use working tools

The participants reported that they sometimes struggled to use their working tools due to the nature of their disability, which affected their performance. This was explained by a 27-year old man (M) with lame left limbs and learning disability:

‘I do struggle to sew due to my disability, the machine is faster than me, and I am very slow’.

This was also corroborated by another 28-year old man (F) with bone and joint deformities and visual impairments:

‘Problem I get is not getting the beads that I like because I cannot see properly’.

Some of the participants stated that they encountered some difficulties when using scissors and measuring.

This barrier was also detected during observations, as some of the participants were unable to use the tools correctly. For example, participants in the beading project were struggling to use the fish line, which is a tool used to pick up beads. Some of the participants who were involved in a knitting and crochet work project struggled to hold crocheting hooks while others did not know how to use scissors.

Interpretation of these findings indicates that people with disabilities’ working equipment are not adjusted and modified to accommodate the needs of the participants despite the mandate of Code of Good Practice (Republic of South Africa 2002). This is similar to a study on skills development for youth living with disabilities in four developing countries (Kett 2012). Also, to support these findings, scholars (Berthoud 2008; Jones & Jones 2008; Meager & Higgins 2011) state that, different impairment characteristics, as well as the type, severity and health problems, influences the participation rate of people with disabilities. The social model argues that it is the society that impairs the individuals, in this case the society has failed to provide adequate accommodations to the participants.

#### Inability to concentrate on the activities for long periods

Inability to concentrate on activities for long periods was observed and highlighted in the interviews as one of the barriers that hindered participation. Some participants, for example, indicated that they got easily distracted and could not focus for long and other participants stated that they got bored of doing the same thing. This is similar to the findings of the working paper on livelihood challenges for extremely poor disabled people in the southwest coastal region of Bangladesh (Nokrek, Alam & Ahmed 2013). A 26-year old woman (G) with spina bifida stated:

‘I cannot concentrate because I get tired easily’.

#### Lack of funds

The issue of funding was another barrier faced by the participants. All the participants highlighted that their biggest challenge was lack of funds to purchase appropriate materials. This was evident as some of the participants were using tiny sized beads, which were donated by community members. The participants indicated that the beads they were working with were the only ones available, and this hindered their full participation. Additionally, the participants reported that they were no longer receiving a stipend due to lack of production caused by lack of funds. To support the general view of the participants, the following was captured from a number of transcripts:

‘There is no money here to buy proper material, they give us scraps to work with, and we have not been paid for months now’.

Another 44-year old woman (I) with involuntary muscle movement in both legs stated:

‘I do not like working with different textures; some textures tangle, I so wish they can get proper material that does not tangle, but we were told that there is no money. I cannot proceed with my work the way I would like to because I need to keep on fixing the wool. It is a waste of time and energy, and it is frustrating me’.

Lack of funds continues to be a challenge for people with disabilities and is a barrier to participation. This challenge resulted in participants having to use inappropriate material for their projects, which in return resulted in poorly constructed products. This hindered the growth of their projects. Kaeane and Ross (2014) and Schneider (2006) also reported that limited funding hindered the ability of projects to grow. Lack of non-human resources specifically funds, were frequently mentioned to be barriers in a study by Hästbacka, Nygård and Nyqvist (2016). This lead to increased demands for cost-effectiveness, as well as demands for eliminating unnecessary and costly obstacles in service provision. It is also evident from the findings that generally, programmes for the empowerment of people with disabilities were underfunded or not funded at all. This finding is supported by White Paper on the Rights of Persons with Disabilities (Republic of South Africa 2017), which indicates that one of the weaknesses in the subsidised supported employment environment that offers income generating activities opportunities to their members is the lack of financial support. Non-payment of stipends is a serious concern because it has a direct influence on the motivation of the participants (Lack of motivation is another barrier addressed in this section). Remuneration plays a role in attracting and retaining individuals (Mabaso & Dlamini 2017) and could be a reason for participants’ lack of motivation and dissatisfaction.

#### Language barriers

This barrier was an issue of concern. Participants pointed out that they were experiencing a language barrier with the facilitator because she only spoke English and Afrikaans. The participants spoke mostly Sesotho and isiXhosa and did not understand English due to their low educational achievement. This had a negative impact on their participation, as they could not effectively communicate with the facilitator. This barrier, according to the participants, hindered their participation as they were unable to explain what they wanted to do. This point was aired by a 38-year old woman (P) with Down syndrome:

‘I am reluctant to seek help because I do not know how to speak English. I only know how to speak Sesotho, so I end up doing nothing’.

The participants’ views are supported by Kamil (2015), who contend that communication restriction inhibits learning to progress successfully and lack of ability to communicate in English can lead to communication barriers. A similar finding was also indicated by Gunatilaka et al. (2010). This finding indicates that workplace accommodation guideline (providing interpreters for people with disabilities) stipulated in the Code of Good Practice (Republic of South Africa 2002) is not being followed by the organisation.

#### Lack of motivation

The participants cited the lack of motivation to produce either new products or good quality products as another challenge they experienced. Participants felt that they were doing the same activities all the time, and when they finished their tasks, they did not receive appreciation and positive recognition. This point was made by a 28-year old man (F) with bone and joint deformities.

‘There is nothing new from what I knew then and now, whatever I learnt and knew is from my previous school. I got demotivated here; we do the same boring things every day, I wanted to do welding, wood hangers and key holders’.

A 46-year old man (Q) left paralysed by a stroke in his left arm also stated:

‘We do not have deadlines, no motivation from the facilitators to produce good quality products’.

The general picture that emerges from this is that some of the participants’ needs are not met, hence they are demotivated. It also appears that intrinsic motivation such as to use one’s ability, receiving appreciation, positive recognition, as well as being treated in a caring and considerate manner seems to play a crucial role in motivating the participants. According to Joseph (2015), individuals have habits and needs that must be managed in order to be motivated. This aspect together with the language barrier to communication should be crucial in the management of people with disabilities.

#### Activities that are not stimulating

Some participants claimed that some of the projects they were taking part in were not stimulating. As a result, they ended up wandering around or not finishing their work. This was pointed out by a 28-year old man (F) with bone and joint deformities and visually impaired who stated:

‘I do not enjoy what I am doing because it does not challenge me and I am not interested in what they have here, so I end up not finishing what I am supposed to do’.

This was also corroborated by another 24-year old man (H) with cerebral palsy:

‘This place does not a have a long-term plan and a clear vision for me, I have lots of things I want to pursue I want to concentrate on business’.

The views are supported by Brouwers, Van Brakel and Cornielje (2011), who contend that people with disabilities involved in low skilled jobs offer little or no opportunities for career progression and are condemned to produce homemade products which are uninspiring. Non- stimulating activities was reported as a barrier in a study by Naami (2014). This finding accentuates the need for the development of more modern market-driven projects that will generate income for the participants.

#### Lack of artistry skills

Artistry refers to the quality imparted to a product in the process of making as well as a human attribute relating to knowledge and skill at performing a task. During observations, the researcher noticed that some of the projects were constructed in such a way that there was no prospect for growth, neither was there any useful structure for a long-term vision for the projects. Some of the products, for example, knitting, tapestry and beading, were constructed unevenly and there were inconsistencies in the construction of the patterns. It was also observed that some participants did not measure the material before cutting and as a result, ended up with yarn being wasted. One participant was making a beaded necklace that was uneven because the participant did not count the beads. Therefore, she had to redo it in order to make it wearable. The root cause of this barrier is low skills levels of the participants, which may be due to lack of basic education and training and that the quality of training received by the participants was poor. Lack of skills amongst people with disabilities has been identified as a major challenge, which inhibits their economic participation (Momin 2004; Schneider 2006; Soudien & Braxton 2006). Powers (2008) points out that compared to people without disabilities, people with disabilities exhibit a skills deficit, which interacts with other barriers to create a double disadvantage in the labour market. The strategy to overcome this hurdle would be to provide various basic quality training, including information and communication technology skills (ICT) to people with disabilities.

UNESCO (2009) asserts that education and training will empower the disadvantaged to take control of their lives. Development of relevant skills is the key to improving productivity and can open doors to economic participation.

### Attitudinal barrier

Exclusion from decision making was one significant attitudinal barrier that hindered the participation of people with disabilities.

Participants felt that they were not taken seriously and were excluded in decisions taken by either the supervisors or management. This was pointed out by a 29-year old woman (A) with epilepsy:

‘They do not take us seriously, and they do not trust us to make our own decision the supervisor chose which colours to use for our products’.

This was also corroborated by another 43-year old woman (D) with a learning disability:

‘Things change here all the time without being informed’.

The fact that some of the participants could not choose the colours and activities to work on, while others got to choose might have been due to the misconception about the ability of people with disabilities to perform well in activities. This view confirms Naami’s (2014) statement, that people with disabilities experience discrimination in all spheres of daily life. Naami (2010) mentions in another study the negative impact of unemployment on women with physical disabilities in Tamale, Ghana. Carney (2002) further points out that being excluded in decision making is typical among the worlds’ vulnerable people. The participants’ view is also supported by Moran, Gibbs and Mernin (2017), who indicate that individuals with disabilities often experience very little control over their own lives as decisions are made for them. The involvement in decision making by people with disabilities is included in the guidelines for the integration of people with disabilities as set out in the White Paper for Social Welfare (Republic of South Africa 1997a).

The examples mentioned above indicate that these barriers can lead to the exclusion of people with disabilities. Hence, the perspective of the social model highlights that disability is not only an individual medical issue rather when changes are made in society, then people with disabilities can be included.

### Coping strategies used to overcome the barriers

Some of the participants shared positive stories of what worked well for them despite the challenges. Guidance from co-workers, experience from a previous school and improvisation were reported.

#### Guidance from co-workers

Some of the participants reported that they overcame the language barrier by asking one of the co-workers who were multilingual to translate for them. This point was aired by a 43 year old man (D) with stroke on the right side.

‘When I do not understand what is being said by the manager or facilitator, I usually ask my friend who speak English and Sesotho to help me, and I do not know what I would have done if it wasn’t for him because I only speak Sesotho’.

This indicates an example of the value of teamwork and that accommodations need not to be expensive.

#### Experience from a previous school

One of the participants who assisted the facilitator with IGAs reported on how he overcame the struggle of not being able to use the working tools properly and how he assisted other participants who struggled to count and use the tools. The following excerpt by a 24-year old man (H) with cerebral palsy briefly describes how he used his experience to help his co-workers:

‘I overcame my struggle by first drawing and writing on the paper, what I am supposed to do before I knot the fishline. I then practise and practise until I get it right, and that works well. I now use the same method when I struggle with my work, and when I teach my colleagues’.

The participant reported that he learnt this method from his previous school. The participant’s view is confirmed by Block, Taliaferro and Moran (2013), who contend that peer tutors serving as a ‘workbuddy’ are helpful and can be used to motivate participants when there is a shortage of staff.

#### Improvisation

The participants indicated that they used the material that was available and used their creativity to carry on working.

These findings indicate that the participants were able to use their problem-solving skills to remove some of the barriers. Solving problems (use of available resources and recycling material) and seeking support and guidance from peers in order to participate conclusively, demonstrate the interactional and behavioural components of empowerment (Zimmerman 1995, 2000).

## Conclusion

This study illustrated the barriers faced by people with disabilities participating in IGAs in a sheltered workshop. The participants reported on the challenges, which were mostly institutional. Such barriers limited their economic participation. The understanding of barriers to economic participation for people with disabilities gained through this study is essential for the effective intervention and development of strategies to promote their participation. It is noted that all these challenges experienced, directly and indirectly, influenced the participation in the projects and some were beyond the scope of a person to change.

Institutional barriers have various consequences for people with disabilities. The social model of disability suggests that the collective disadvantage of people with disabilities is due to a complex form of institutional discrimination. The social model also purports that modern-day discrimination is a socially created phenomenon, which in reality has little to do with the actual physical or mental impairments of disabled people. Thus in the case of this study, the cure lies in restructuring society by addressing workplace accommodation.

### Limitations

This study took place in one sheltered workshop. The sample size did not allow for the generalisation of results, although data were collected until saturation was reached. The views of the participants in the sample do not represent the views of all people with disabilities participating in IGAs. The study only focused on people with disabilities.

### Recommendations

There is a need for good basic education to ensure follow-through with skills and employment. Improved existing training programmes that accommodate the needs and challenges of people with disabilities are required because training can lead to better outcomes and improved output. The training programmes may include vocational education and skills training, offered as short courses will enable people with disabilities as well as facilitators to acquire adult basic education and skills that are relevant to the current labour market demands. The organisation could also meet the needs of people with disabilities by providing supported employment programme to prepare those who want to enter the job market. Part of training programmes could also include diversity and inclusion workshops whereby a specialist facilitator will visit the sheltered workshop and present sessions with support workers and people with disabilities. Such workshops can help build skills, such as communicating better with people from diverse backgrounds and reducing the levels of unconscious bias in their decision-making. Methods of communication such as sign language and interpreting services, speech software and Braille printing should be considered. Support workers should be trained in Sesotho and isiXhosa in order to communicate with people with disabilities. It is also suggested that the government should improve funding for IGAs and that funders of income-generating projects should form part of a future study. Facilitators could be included in future research as their viewpoints would provide further depth and ensure another dimension to the data collected. Seeing that coworkers can assist in overcoming some of the barriers experienced it is therefore recommended that workers who speak the same language could be positioned next to each other. Use of peer tutoring with adequate knowledge and skills could also make a difference in the lives of other peers in a number of ways.
